# Evaluation of an artificial intelligence support system for breast cancer screening in Chinese people based on mammogram

**DOI:** 10.1002/cam4.5231

**Published:** 2022-09-09

**Authors:** Chengzhen Bao, Jie Shen, Yue Zhang, Yan Zhang, Wei Wei, Ziteng Wang, Jia Ding, Lili Han

**Affiliations:** ^1^ Beijing Obstetrics and Gynecology Hospital Capital Medical University. Beijing Maternal and Child Health Care Hospital Beijing China; ^2^ Yizhun AI Company Beijing China

**Keywords:** artificial intelligence, AUC, breast cancer, sensitivity, specificity

## Abstract

**Background:**

To evaluate the diagnostic performance of radiologists on breast cancer with or without artificial intelligence (AI) support.

**Methods:**

A retrospective study was performed. In total, 643 mammograms (average age: 54 years; female: 100%; cancer: 62.05%) were randomly allocated into two groups. Seventy‐five percent of mammograms in each group were randomly selected for assessment by two independent radiologists, and the rest were read once. Half of the 71 radiologists could read mammograms with AI support, and the other half could not. Sensitivity, specificity, Youden's index, agreement rate, Kappa value, the area under the receiver operating characteristic curve (AUC) and the reading time of radiologists in each group were analyzed.

**Results:**

The average AUC was higher if the AI support system was used (unaided: 0.84; with AI support: 0.91; *p* < 0.01). The average sensitivity increased from 84.77% to 95.07% with AI support (*p* < 0.01), but the average specificity decreased (*p* = 0.07). Youden's index, agreement rate and Kappa value were larger in the group with AI support, and the average reading time was shorter (*p* < 0.01).

**Conclusions:**

The AI support system might contribute to enhancing the diagnostic performance (e.g., higher sensitivity and AUC) of radiologists. In the future, the AI algorithm should be improved, and prospective studies should be conducted.

## Graphical abstract

The average AUC was significantly increased from 0.84 to 0.91, average sensitivity significantly increased from 84.77% to 95.07%, and average specificity decreased but no statistical difference was observed when reading with AI support. The average reading time significantly decreased from 215 s per mammogram to 106 s per mammogram when using AI support system.

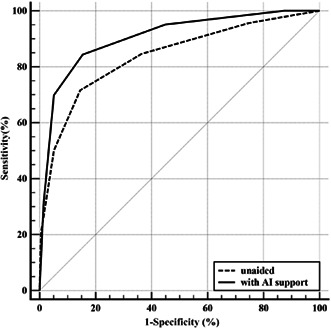

## 1 INTRODUCTION

Female breast cancer is the most commonly diagnosed cancer worldwide, and approximately 685,000 patients die of breast cancer every year.^1^ Early detection of breast cancer improves treatment and reduces the burden of disease^2^; thus, screening programs have been conducted in some countries (e.g., China). Compared with Asia, the incidence rate of breast cancer was higher in Europe,^3^ and a previous study found that, if the examination coverage reached 100% in all European countries, then more than 12,000 additional breast cancer deaths could be avoided each year.^4^ At least one radiologist is required to assess mammograms per screening test, and the workload of each radiologist could be higher if examination coverage increased. Artificial intelligence (AI) innovations in radiology might be a solution to workforce shortages, and the increasing need for screening might be satisfied.^5^, ^6^

AI is a branch of computer, and AI‐based algorithms have been designed and improved to simulate human intelligence, such as the ability to diagnose diseases. Several AI software algorithms used to recognize breast cancer have been developed,^7^, ^8^ and compared with radiologists, some of them showed similar or better performance (e.g., higher sensitivity and specificity) in assessing mammograms.^9^, ^10^ In a previous study of likely negative mammograms, most primary care providers accepted the use of AI to make decisions without radiologist confirmation,^11^ which might be beneficial to expand the coverage of screening prior to solving the shortage of radiologists. However, AI software still has certain limitations, such as temporal comparison and symmetry comparison.^12^ Additionally, social consensus is needed for the use of AI algorithms on mammography interpretation,^13^ and issues about imputation of responsibility (e.g., radiologist, machine and builder of the AI tool) need to be solved if the autonomous determination of AI algorithms is wrong.^14^ Moreover, the false positive and false negative were still inevitable, which might result in the waste of medical resources or covering up the illness.

This study aimed to explore the probability of using AI as a read aid in breast cancer screening by analyzing the diagnostic performance of radiologists on breast cancer with or without AI support in Chinese cases. The findings might contribute to the continual improvement of breast cancer screening programs in China.

## 2 METHODS

This retrospective study was conducted in accordance with the Ministry of Science and Technology of the People's Republic of China. The medical ethics committee of Beijing Obstetrics and Gynecology Hospital, Capital Medical University Beijing Maternal approved this study. Data were collected from two 3A hospitals and one tertiary hospital in China. The inclusion criteria were as follows: (1) the format of the images was Digital Imaging and Communications in Medicine (DICOM); (2) a lesion could be found in the image; (3) with initial Breast Imaging Reporting and Data System (BI‐RADS). The exclusion criteria were as follows: (1) low‐quality mammogram; (2) less than two views (e.g., left craniocaudal view, left mediolateral oblique view, right craniocaudal view, right mediolateral oblique view) composed each mammogram; (3) lesion not identifiable by mammogram and other examinations were necessary (e.g., ultrasound, magnetic resonance imaging); (4) without biopsy result. Mammograms were divided into two groups by stratified randomization based on initial BI‐RADS. Both groups were assessed by radiologists, and one of them was initially assessed by AI. Radiologists judged whether they accepted the advice of AI regarding every detail (e.g., breast density, lesion type). In consideration of the differences in capability among radiologists, 75% mammograms in each group were selected by stratified sampling based on initial BI‐RADS and were read by any two radiologists independently.

Each radiologist was blinded to any information (e.g., age, sex, biopsy result) related to the person whose mammogram was assessed. After random allocation, half of them read with AI support, but the mammograms they read were randomly selected. Before reading, every radiologist was trained to familiarize themselves with the evaluation criteria and virtual workstation, and those who were supported by AI familiarized themselves with the AI support system. For each mammogram, the radiologist recorded the following information: (1) BI‐RADS score from 0 to 5, with BI‐RADS 4 including 4A, 4B and 4C; (2) lesion type, including mass, calcification, asymmetric densification, and structural distortion; (3) breast density, including fatty, mildly dense, moderately dense, and dense.^15^

The AI support system made by Yizhun AI company was a mammogram auxiliary diagnostic software (version 3.2.3, Yizhun AI). The system was mainly based on the deep neural network model (Figure S1 in supporting information); it indicated the area, category and BI‐RADS of suspected lesions to radiologists to effectively enhance the sensitivity and prevent missed diagnoses in breast cancer screening (Figure S2 in supporting information). The system included the following five modules: (1) The feature pyramid network extracted multiscale feature maps from input images. (2) The feature combination network realized the left and right feature fusion of mammograms, as well as craniocaudal and mediolateral oblique view feature fusion. This module simulated the diagnosis experience of doctors to improve the accuracy of lesion detection. (3) The region of interest (ROI) extraction network selected the region of interest from the anchor, which may contain the lesion. (4) The ROI classification and regression network further determined whether the region of interest contained lesions and outputted the lesion type and bounding box. (5) The contours of the detected lesions were outputted through the lesion segmentation network. Doctors could use the contours to measure the long and short diameters of the lesions and write a report.

The system was trained and validated based on a database containing more than 16,000 mammograms with different types of lesions (one‐third of which were calcifications and masses) and normal mammograms, and the mammograms for training and validating originated from devices produced by many different vendors (GE, Philips, Siemens, Hologic, etc.). The system was tested on an independent internal multivendor dataset that had not yet been used for algorithm training or validation. The mammograms used in this study had never been used to train, validate, or test algorithms.

The system covered the detection of almost all types of disease in mammography, including mass, calcification, asymmetric densification, structural distortion, axillary lymphadenopathy, skin retraction, skin thickening, nipple retraction, as well as predicting gland types and BI‐RADS. The system simulated the doctor's diagnosis rules and film reading experience and simultaneously added an attention mechanism to fuse information from the craniocaudal and mediolateral oblique views of both breasts to achieve the precise detection and diagnosis of lesions. The radiologist used an interactive decision while using this system for support in practice. The system gave a list of lesions corresponding to the current case. The radiologist clicked on the list of lesions to display the contour of the current lesion and its corresponding diagnostic information. If radiologists reread mammograms based on the evaluation of this system, they could move, zoom and flip the image; adjust the window width and position of the image; and measure the size of the lesion through the ranging function. After rereading, the radiologist revised the features of the lesion and BI‐RADS if necessary.

The diagnostic performance was comprehensively analyzed with the sensitivity, specificity, Youden's index, agreement rate, Kappa value, area under the receiver operating characteristic (ROC) curve (AUC) and reading time of the radiologists. Statistical analysis was performed with SAS, SPSS, and MedCalc software. T tests, Wilcoxon rank sum tests, or chi‐square tests were conducted to analyze the differences in distribution between two groups. *p* < 0.05 was regarded as statistically significant.

Sensitivity, specificity, Youden's index, agreement rate, Kappa value and AUC were computed by BI‐RADS of the American College of Radiology and biopsy result (gold standard). BI‐RADS 1–3 indicated benign, and BI‐RADS 4–5 were classified as malignancy in this study.^15^ If some radiologists classified the mammogram as BI‐RADS 0, which meant that lesions could not be identified by mammogram and other examinations were necessary, the records could not be included in the comparison study.^15^ The reading time per mammogram was automatically calculated by the virtual workstation, which was the time interval between when records were successfully submitted and image loading was completed.

## 3 RESULTS

Overall, 643 mammograms were collected (average age: 54 years; female: 100%; cancer: 62.05%), and half of them (321 mammograms) was initially assessed by AI. And 75% mammograms in each group were read by two radiologists, so 564 unaided assessments and 562 assessments with AI support were conducted in the two groups (Figure S3 in supporting information). Two records in both groups were BI‐RADS 0; thus, 1122 records (560 records were assessed with AI support) were statistically analyzed. The characteristics of mammograms in the two groups are shown in Table 1. There were no significant differences in the distributions of breast density and biopsy results between the two groups. The characteristics of mass, calcification, and structural distortion were significantly different. Seventy‐one radiologists who had experience assessing mammograms participated in this study, and half of them (35 radiologists) read with AI support. The average age among the radiologists was 38 years, and the average years of experience as a radiologist was 5 years. The majority of them (74.65%) were female, and more than a third of them (39.44%) were master. As Table 2 reports, there were no significant differences in age, sex, education or radiologists' years of experience for radiologists between the groups.

**TABLE 1:** Characteristics of mammograms in two groups

Variable	Unaided		With AI support		*p* value
	*N*	%	*N*	%	
Breast density					0.13
Fatty	81	14.41	62	11.07	
Mildly dense	205	36.48	145	25.89	
Moderately dense	188	33.45	319	56.96	
Dense	88	15.66	34	6.07	
Mass					<0.01
Yes	354	62.99	463	82.68	
No	208	37.01	97	17.32	
Calcification					<0.01
Yes	234	41.64	293	52.32	
No	328	58.36	267	47.68	
Asymmetric densification					0.15
Yes	58	10.32	44	7.86	
No	504	89.68	516	92.14	
Structural distortion					<0.01
Yes	37	6.58	8	1.43	
No	525	93.42	552	98.57	
Biopsy result					0.91
Cancer	348	61.92	345	61.61	
Nomal	214	38.08	215	38.39	

**TABLE 2:** Characteristics of radiologists in two groups

Variable	Unaided		With AI support		*p* value
	*N*	%	*N*	%	
Age					0.62^a^
<35 years	11	30.56	12	34.29	
≥35 years	25	69.44	23	65.71	
Sex					0.94^b^
Male	9	25.00	9	25.71	
Female	27	75.00	26	74.29	
Education					0.53^b^
Bachelor or below	20	55.56	22	62.86	
Master or above	16	44.44	13	37.14	
Radiologists' years of experience					0.83^a^
<5 years	16	44.44	17	48.57	
≥5 years	20	55.56	18	51.43	

^a^ Wilcoxon rank sum test.

^b^ Chi‐square test.

**FIGURE 1:**
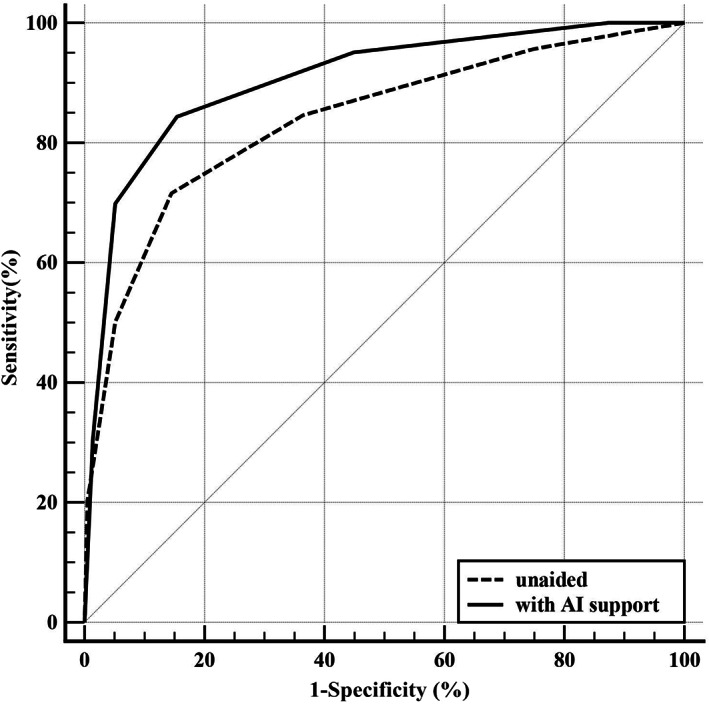
Average receiver operating characteristic (ROC) curves of all radiologists in two groups

**FIGURE 2:**
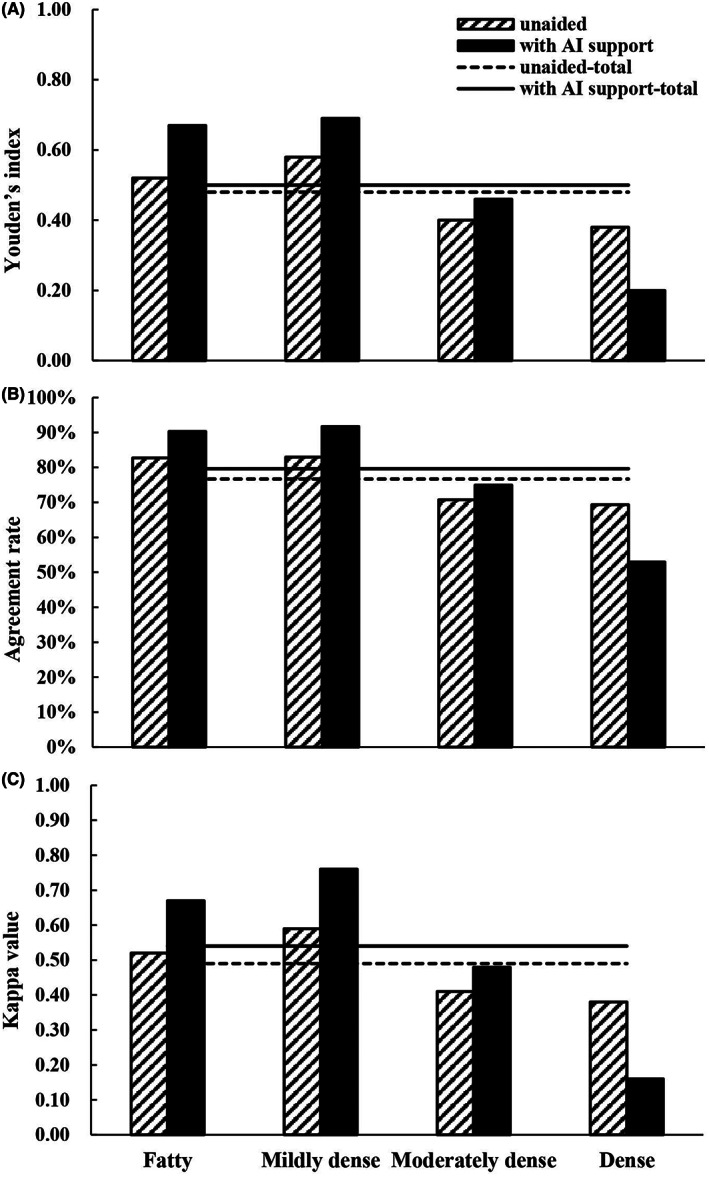
Youden's index (A), agreement rate (B), Kappa value (C) of all radiologists in two groups

Assessing with AI support was useful for improving the performance of radiologists in this study (Figure 1, Figure 2, Table 3), and the average AUC was increased from 0.84 to 0.91 (*p* < 0.01). In all subgroup scenarios, except for dense cases, the AUCs were larger with AI support, but significant differences could be found only in moderately dense cases (*p* < 0.01). Average sensitivity significantly increased from 84.77% to 95.07% when reading with AI support (*p* < 0.01). As Table 3 shows, the sensitivity was larger with AI support in each subgroup, and the improvement was significant in the mildly dense (*p* < 0.01) or moderately dense (*p* < 0.01) cases. On average, specificity decreased when using the AI support system, but no significant differences were observed (*p* = 0.07). In the two low density subgroups, that is, fatty and mildly dense cases, specificities were increased to varying degrees if the AI support system was used, and the other two subgroups were opposite. In addition, there was no significant difference in specificity in each subgroup. In terms of Youden's index, agreement rate and Kappa value, the averages of all cases that were assessed with AI support were larger and could also be found in subgroups except for dense cases (Figure 2).

**TABLE 3:** Performance of screening in two groups

Variable	Unaided	With AI support	*p* value
	Value (95%CI)	Value (95%CI)	
Sensitivity (%)			
Total	84.77 (80.46, 88.29)	95.07 (92.08, 97.01)	<0.01^a^
Fatty	88.71 (77.51, 94.96)	94.12 (82.77, 98.47)	0.50^a^
Mildly dense	90.00 (83.49, 94.22)	99.07 (94.20, 99.95)	<0.01^a^
Moderately dense	77.23 (67.61, 84.74)	93.68 (88.68, 96.64)	<0.01^a^
Dense	80.00 (64.95, 89.91)	83.33 (50.88, 97.06)	0.90^a^
Specificity (%)			
Total	63.55 (56.68, 69.93)	54.88 (47.97, 61.62)	0.07^a^
Fatty	63.16 (38.63, 82.77)	72.73 (39.32, 92.67)	0.70^b^
Mildly dense	67.69 (54.82, 78.46)	70.27 (52.83, 83.56)	0.79^a^
Moderately dense	63.22 (52.15, 73.11)	52.41 (43.99, 60.71)	0.11^a^
Dense	58.14 (42.21, 72.63)	36.36 (18.03, 59.17)	0.10^a^
AUC			
Total	0.84 (0.81, 0.87)	0.91 (0.88, 0.93)	<0.01^c^
Fatty	0.89 (0.81, 0.96)	0.94 (0.88, 1.00)	0.29^c^
Mildly dense	0.90 (0.85, 0.94)	0.96 (0.91, 1.00)	0.05^c^
Moderately dense	0.78 (0.72, 0.84)	0.90 (0.87, 0.94)	<0.01^c^
Dense	0.75 (0.65, 0.85)	0.71 (0.53, 0.89)	0.68^c^

^a^ Chi‐square test.

^b^ Fisher's exact test.

^c^ Z test.

The average reading time was significantly different (*p* < 0.01) between the unaided group (median: 215 s per mammogram) and the group with AI support (median: 106 s per mammogram). The findings in subgroups classified by biopsy result were similar (cancer: *p* < 0.01; normal: *p* < 0.01). In the unaided group, the average reading time of cancer (median: 221 s per mammogram) was larger than that of all cases, but the opposite was true in the group with AI support (median: 101 s per mammogram). In normal persons, the difference in average reading time between the two groups (unaided: median = 202 s per mammogram; with AI support: median = 112 s per mammogram) was less than that in all cases.

## 4 DISCUSSION

In this study, the average diagnostic performance of the radiologists who assessed mammograms with AI support was higher than those who assessed unaided mammograms (as shown by the AUC). In moderately dense cases, the superiority of diagnostic performance on breast cancer by using AI as a reader aid was significant (AUC: *p* < 0.01). The average reading time declined with AI support, especially in confirmed breast cancer, which was to the benefit of reducing the time cost of screening. Meanwhile, compared with the unaided group, significantly higher sensitivity and no statistically lower specificity were observed in the group with AI support. Therefore, using AI as reader aid had the advantage of increasing the effectiveness of breast cancer screening. However, the overdiagnosis issue (increase of 8.67% in false positive) still should be paid more attention to by reason of the borderline significance on specificity (*p* = 0.07) in this study, which might lead to the waste of medical resource and additional pain to the patient.

The AI algorithm and representativeness of the training sample influenced the diagnostic performance. In this study, the AUCs did not differ between the groups regarding fatty and dense cases, which might be attributed to limited training samples (e.g., small sample size, similar characteristics of sample). In addition, in terms of dense cases, it was more difficult to differentiate lesions from normal tissue based on the existing algorithm, which could also reduce the sensitivity and specificity of the AI support system. It was reported that in China more than 80 percent of breast cancer patients were mildly (33.07%) or moderately (48.29%) dense cases,^16^ so if the AI support system was used in screening, more positive cases might be found. To enhance the applicability in breast cancer screening, the AI support system should be improved by increasing the variety and quantity of training samples, especially mammograms of fatty and dense cases. In addition, the algorithm should be optimized so that the capability of identifying lesions could be improved.

AI techniques for breast imaging studies, such as artificial neural networks,^17^ machine learning,^18^ and deep learning, had varied.^19^ Some AI tools conducted deep learning with mammograms only, and demographic and clinical data were not used in training sessions, which might have decreased the sensitivity or specificity.^20^ In clinical practice, before drawing a conclusion, a radiologist would conduct a comprehensive evaluation based on the characteristics of mammograms and other risk factors, such as age, past medical history, family history, and estrogen exposure, which was a missing step in the AI algorithm. The challenge for AI was the inclusion of a large number of variables in the training of the algorithm (e.g., age, prior breast cancer, hormone supply treatment) and imitating the radiologist's workflow, which demanded large‐ and high‐quality data.^21^ However, it was also important to avoid overfitting when large and high quality data were included, which prevented poor performance (e.g. sensitivity, specificity or AUC) on testing or validation sets even if good performance on the training set was found.^22^

In this study, the superiority of the diagnostic performance using AI as a reader aid was relatively definite, and the AUC was better than that in other similar studies.^23^, ^24^ In addition to using AI as a reader aid, some studies used AI for triage prescreening (radiologists assessed after removing normal cases by AI)^6^, ^25^ or used AI as a standalone system (radiologists were replaced by an AI system).^26^, ^27^ The sensitivity and specificity of AI systems were variable in previous studies and unsatisfactory in some studies.^6^, ^23^, ^24^, ^25^, ^26^, ^27^ It was not clear how AI complemented the clinical pathway to maximize performance and which radiologists had greater demands for AI support systems. In this study, the average reading time significantly declined with AI support; thus, the indirect cost might have been lower. However, the cost of purchasing an AI support system should also be taken into consideration. Moreover, some hospitals might need to replace hardware before using AI support systems. Therefore, based on the principle of maximum benefit, economic evaluation of AI support systems in breast cancer screening should be conducted in the future.^28^

Some previous studies defined “non‐cancer” as a negative diagnosis at follow‐up for several months,^23^, ^24^, ^29^ which was more likely to find cancer from the original negative result. The final characteristics of a mammogram at follow‐up might be different from those of the initial mammogram. Therefore, in the retrospective study, only mammograms in the last screening were included in for analysis. To enhance the performance of the AI support system in forecasting, a prospective cohort study can be conducted, which focuses on patients with negative biopsy results. Based on several regular mammograms during the follow‐up period, the mammograms for training can be enriched, and the AI algorithm can be optimized, which may be beneficial for the early identification of suspicious breast cancer.

There were some limitations in this study. First, as a retrospective study, the percentage of cancer in subjects was larger than that in the real world; thus, external validation in a clinical cohort or real screening might be needed.^30^ Second, the bias from the subjective preferences of radiologists could not be estimated. When the advice of AI was right, some overconfident radiologists might be opinionated even if they were erroneous. Conversely, those who lack self‐confidence might be overreliant on the AI support system when they disagree over the diagnosis. Third, Chinese patients have higher breast density than other ethnicities,^31^ but the AUC on dense breasts did not increase by using AI as a reader aid. A previous study reported that high breast density was an independent risk factor for breast cancer,^32^ and dense tissue might mask lesions and lead to false negative increases. In the future, the AI algorithm should be improved by further training. Finally, some characteristics of mammograms were unevenly distributed between the two groups (e.g., mass, calcification and structural distortion), which might have led to selection bias, so more proper randomization (e.g. stratified randomization based on category and BI‐RADS of suspected lesion) should be conducted in the further study.

## 5 CONCLUSION

There is considerable interest in using AI for reading mammograms, and a large number of retrospective case–control studies or cohort studies have been reported. More superior diagnostic performance (e.g. higher sensitivity and AUC) by using AI as a reader aid was found in this study, but not all kinds of breast density were suitable for assessment with the current AI support system (e.g., dense breasts). Before the AI support system is used to complement the work of radiologists in Chinese breast cancer‐screening programs, the AI algorithm should be improved, and prospective studies in representative samples of target populations are needed.

## AUTHOR CONTRIBUTIONS

**Chengzhen Bao:** Formal analysis (lead); writing – original draft (lead); writing – review and editing (lead). **Jie Shen:** Data curation (equal); project administration (equal). **Yue Zhang:** Data curation (equal); project administration (equal). **Yan Zhang:** Conceptualization (supporting); methodology (supporting). **Wei Wei:** Data curation (supporting); project administration (supporting). **Ziteng Wang:** Software (lead); writing – original draft (supporting); writing – review and editing (supporting). **Jia Ding:** Conceptualization (lead); funding acquisition (lead); project administration (lead); software (supporting). **Lili Han:** Conceptualization (lead); funding acquisition (lead); project administration (lead).

## FUNDING INFORMATION

This study was funded by Beijing Municipal Science & Technology Commission (No. Z201100005620011).

## CONFLICT OF INTEREST

The authors declare that they have no competing interests.

## ETHICS APPROVAL STATEMENT

This study was approved by Ministry of Science and Technology of the People's Republic of China (No. [2021]CJ0926) and the medical ethics committee in Beijing Obstetrics and Gynecology Hospital, Capital Medical University Beijing Maternal (No. 2020‐KY‐023‐01).

## INFORMED CONSENT STATEMENT

Each participant had sighed informed consent before taking mammogram.

## Supporting information


Figure S1

Figure S2

Figure S3

## Data Availability

The datasets used during the current study are available from the corresponding author.
