# Supplementary figures and images for: Evaluation of an artificial intelligence support system for breast cancer screening in Chinese people based on mammogram
Source: Cancer Med. 2022 Sep 9;12(3):3718–26. doi: 10.1002/cam4.5231 (PMC9939225; doi:10.1002/cam4.5231)

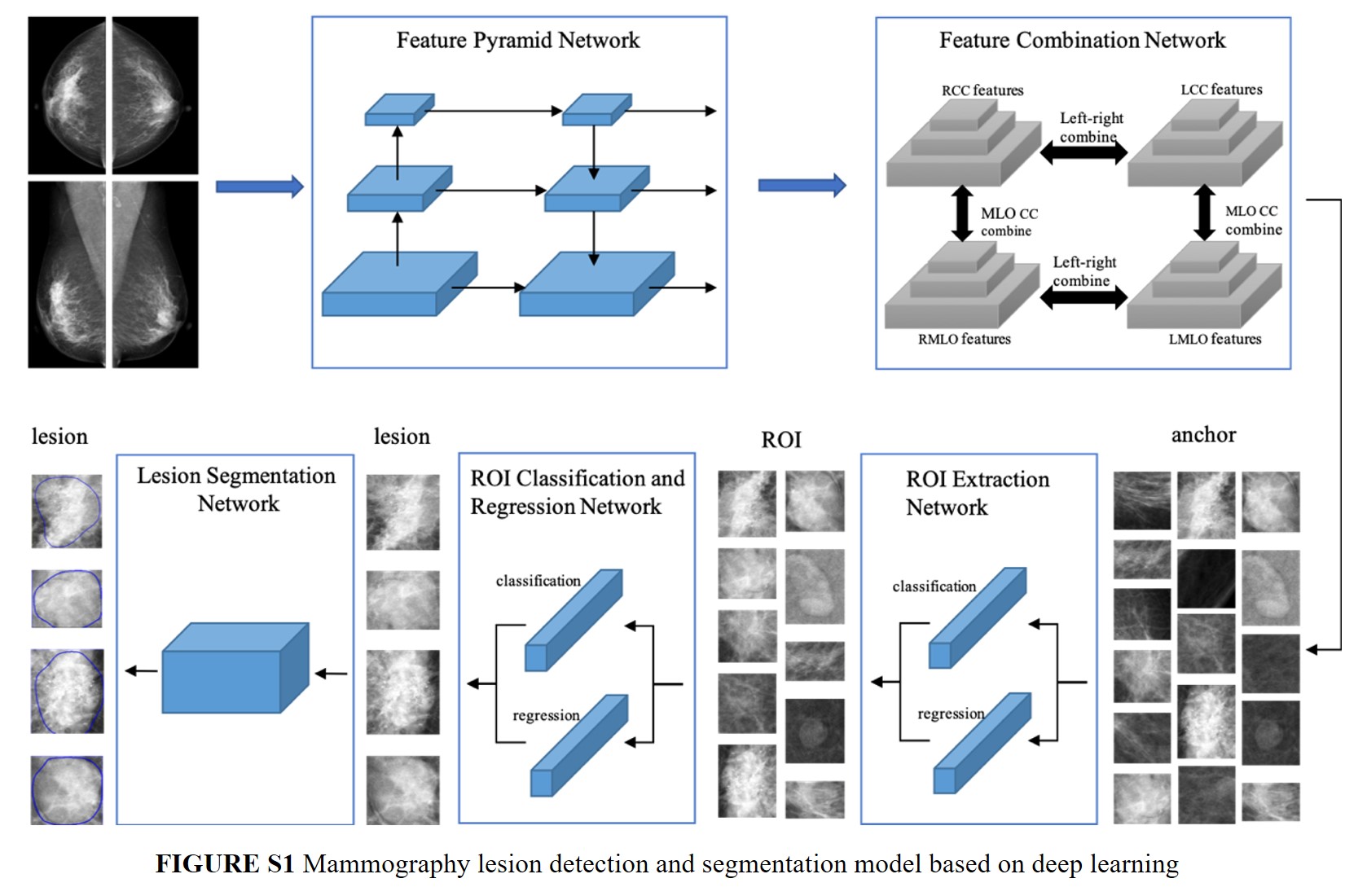

Supplement: Supplementary file 1 — Figure S1 [file CAM4-12-3718-s001.jpg]

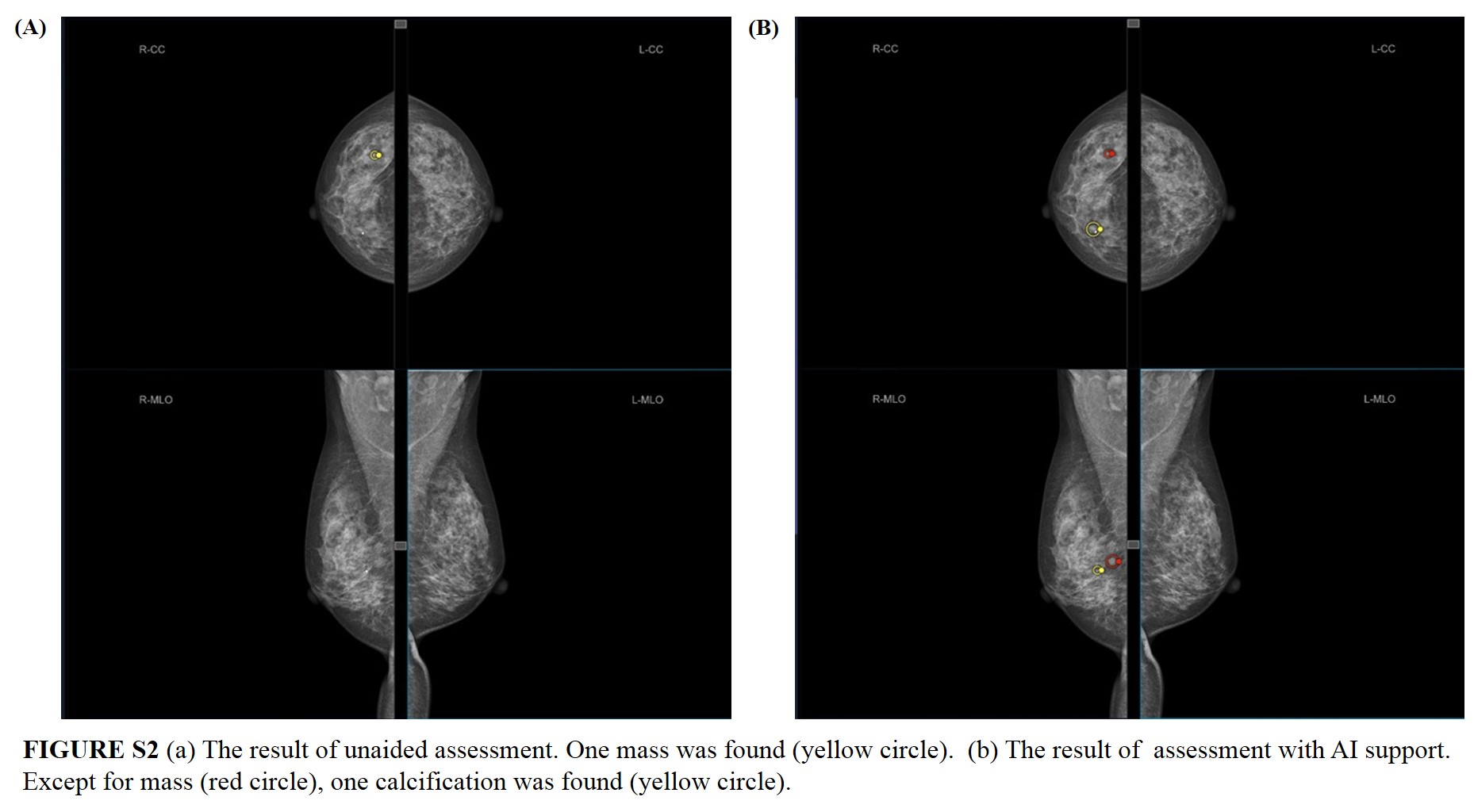

Supplement: Supplementary file 2 — Figure S2 [file CAM4-12-3718-s002.jpg]

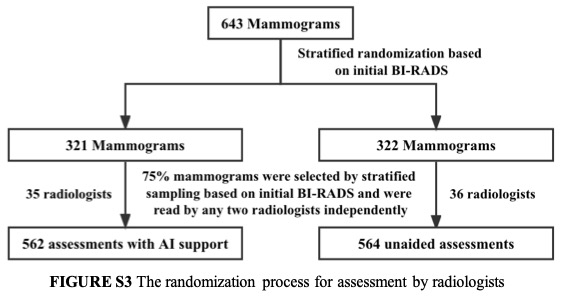

Supplement: Supplementary file 3 — Figure S3 [file CAM4-12-3718-s003.jpg]
